# Epidural Analgesia for Severe Chest Trauma: An Analysis of Current Practice on the Efficacy and Safety

**DOI:** 10.1155/2019/4837591

**Published:** 2019-03-19

**Authors:** Jesse Peek, Reinier B. Beks, B. Feike Kingma, Marije Marsman, Jelle P. Ruurda, Roderick M. Houwert, Loek P. H. Leenen, Falco Hietbrink, Mirjam B. de Jong

**Affiliations:** ^1^Department of Surgery, University Medical Center Utrecht, Utrecht, Netherlands; ^2^Utrecht Traumacenter, Utrecht, Netherlands; ^3^Department of Anesthesiology, University Medical Center Utrecht, Utrecht, Netherlands

## Abstract

**Background:**

Adequate pain control is essential in the treatment of patients with traumatic rib fractures. Although epidural analgesia is recommended in international guidelines, the use remains debatable and is not undisputed. The aim of this study was to describe the efficacy and safety of epidural analgesia in patients with multiple traumatic rib fractures.

**Methods:**

A retrospective cohort study was performed. Patients with ≥3 rib fractures following blunt chest trauma who received epidural analgesia between January 2015 and January 2018 were included. The main outcome parameters were the success rate of epidural analgesia and the incidence of medication-related side effects and catheter-related complications.

**Results:**

A total of 76 patients were included. Epidural analgesia was successful in a total of 45 patients (59%), including 22 patients without and in 23 patients with an additional analgesic intervention. In 14 patients (18%), epidural analgesia was terminated early without intervention due to insufficient sensory blockade (*n*=4), medication-related side effects (*n*=4), and catheter-related complications (*n*=6). In 17 patients (22%), the epidural catheter was removed after one or multiple additional interventions due to insufficient pain control. Minor epidural-related complications or side effects were encountered in 36 patients (47%). One patient had a major complication (opioid intoxication).

**Conclusion:**

Epidural analgesia was successful in 59% of patients; however, 30% needed additional analgesic interventions. As about half of the patients had epidural-related complications, it remains debatable whether epidural analgesia is a sufficient treatment modality in patients with multiple rib fractures.

## 1. Introduction

Thoracic trauma is frequently encountered in the emergency department and is responsible for 10% to 15% of all trauma admissions [1]. Traumatic rib fractures represent an important injury following blunt thoracic trauma and are identified in 10% to 40% of all trauma patients [1–3]. Rib fractures are associated with severe injury and carry a significant morbidity and mortality rate [2, 4]. Factors associated with higher mortality rates are an increased number of rib fractures, advanced age, and concomitant injuries [1, 2, 5, 6]. Furthermore, preexistent (pulmonary) comorbidities have shown to be of significant influence on the outcome [7].

Adequate pain control is key in the management of rib fractures. Pain associated with rib fractures and other thoracic injury can lead to inefficient ventilation resulting in respiratory complications, need for mechanical ventilation, and prolonged recovery [2, 8]. Consequently, multiple analgesic modalities have been described in the last few decades, including epidural catheters, intravenous narcotics, and intercostal, paravertebral, or interpleural blocks. However, epidural analgesia remains the recommended method according to the management guidelines of the Eastern Association for the Surgery of Trauma (EAST) [9].

The use of epidural analgesia remains an important topic of discussion. Over the past decades, several studies reported on beneficial outcomes of epidural analgesia and encouraged the use of epidural analgesia over other analgesic modalities [1, 6, 10–16]. However, there is growing evidence questioning its advantages over other analgesic modalities in the management of severely injured trauma patients [4, 17–19]. Furthermore, the current evidence is of low quality, and therefore, the recommendation of the EAST is conditional [9].

Epidural analgesia may be insufficient due to the high risk of failure and catheter-related problems. In previous studies on the use of epidural analgesia after surgery, failure rates have been reported up to 47% [20]. Furthermore, the use of epidural analgesia is limited by a number of contraindications, such as hypotension and respiration depression, which is even of greater influence on polytrauma patients [21].

Further research on the use of epidural analgesia is needed. There is limited literature regarding the efficacy and complications of epidural analgesia in thoracic trauma. Therefore, the aim of this retrospective cohort study was to describe the efficacy and risk of complications of epidural analgesia for patients with multiple traumatic rib fractures.

## 2. Methods

### 2.1. Study Design and Participants

A single-center retrospective cohort study was conducted in the University Medical Center Utrecht, a level 1 trauma center in the Netherlands. To analyze current practice, all adult patients with three or more rib fractures following blunt chest trauma who were admitted between January 2015 and January 2018 were eligible for inclusion. Patients who received epidural analgesia according to clinical documentation in the electronic patient file were included. Data collection was performed with the use of the Dutch National Trauma Registry, a national prospective database containing all trauma patients admitted to the emergency department in the Netherlands. In multitrauma patients, all concomitant injuries were graded using the abbreviated injury scale (AIS) [22]. Patients were excluded in case the injury with the highest AIS was not located in the thorax since pain control of such injuries cannot be achieved by thoracic epidural analgesia. Other exclusion criteria included the need for immediate mechanical ventilation upon admission and/or transfer to or from another hospital. A waiver of consent was approved by our institutional review board.

### 2.2. Epidural Analgesia Indication and Procedure

According to our hospital's pain protocol, epidural analgesia was indicated for patients with three or more fractured ribs with insufficient pain control despite the use of paracetamol, nonsteroidal anti-inflammatory drugs, and morphine. Epidural analgesia was also indicated in case of an increased risk of respiratory insufficiency due to preexistent comorbidities. Indication for epidural analgesia was made primarily in the emergency department, or secondarily, after admission in the surgical ward. The degree of pain was assessed according to the Numerical Rating Scale (NRS) pain score. The NRS is an 11-point scale to measure pain intensity, ranging from 0 (no pain) to 10 (worst imaginable pain) [23].

Contraindications for epidural analgesia included patient refusal, vertebral fractures, spinal cord injury, traumatic brain injury, Glasgow Coma Scale < 15, unstable pelvic fracture, hemodynamic instability, local infection at the insertion site, or coagulopathy. Epidural catheter placement was performed by anesthesiologists at the level of the thoracic injury. The loss of resistance technique was used to guide a 17-gauge Tuohy needle. After reaching the epidural space, a test dose of 3 ml lidocaine 2% was administered to exclude intravascular or intrathecal positioning. Following appropriate catheter insertion, an initial bolus dose with local anesthetics was administered and a continuous epidural infusion was started with a mixture of bupivacaine 2.5 mg/ml and morphine 0.04 mg/ml. The initial infusion rate was 4 ml/hr. According to the patient's response and degree of pain relief, the infusion rate was gradually increased up to a maximum of 6 ml/kg/h. If the epidural block is still not provided with satisfactory pain relief with sufficient dermatomal coverage despite a maximum administration of epidural analgesia, the epidural mixture was diluted 50% with a mixture of bupivacaine 1.25 mg/ml and morphine 0.02 mg/ml. The maximum infusion rate after dilution was 12 ml/hr. All patients received paracetamol in combination with a nonsteroidal anti-inflammatory drug (e.g., diclofenac or ibuprofen), unless contraindications were present. A urinary catheter was inserted in all cases and remained in place during administration of epidural analgesia.

The NRS scores were measured every 8 hours by the ward nurses. A specialized pain team visited the patients daily to evaluate the adequacy of sensory block, side effects, and complications. Additionally, data regarding catheter placement difficulties, duration of infusion, number of top-ups, need for additional pain medication, reason for epidural catheter termination, and conversion to another analgesic modality were recorded. A top-up was defined as an additional bolus administration of 3 ml lidocaine 2% or bupivacaine 0.25% to provide or restore a sufficient sensory block.

### 2.3. Baseline Characteristics and Outcome Measures

Data were retrieved from a prospective database and completed by checking the electronic patient files. Baseline characteristics included patient demographics (i.e., age, gender, and relevant comorbidities), trauma mechanism, injury severity score (ISS) and AIS scores, concomitant injuries, and rib fracture-related characteristics including number and place of fractured ribs, presence of flail segment, bilateral involvement, dislocation, presence of dorsal fracture, first rib involvement, fractures in upper/middle/lower part of the thorax, and indication for rib fixation. Fracture characteristics were evaluated with the use of computed tomography scans.

The primary outcome measure was the success rate of epidural analgesia during the first 5 days of administration. Successful application of epidural analgesia was defined as follows: (1) sufficient pain control or sensory block and (2) no early termination due to medication-related side effects, or catheter-related complications. Epidural analgesia was also classified as successful in case the catheter was removed within the first 5 days due to satisfactory pain, or if necessary for early mobilization. A distinction has been made between success with or without an additional analgesic intervention. Analgesic interventions included epidural top-up/bolus, adjustment of epidural analgesia, and/or administration of intravenous analgesia. Insufficient pain control was defined, according to the hospital pain protocol, as ongoing severe pain (NRS ≥ 7) with the maximum administration of paracetamol, nonsteroidal anti-inflammatory drugs, and morphine [23]. Insufficient block was defined as any present sensory block that provided insufficient coverage for the corresponding thoracic injury. Minor medication side effects included hypotension, nausea, urinary retention, and pruritus. Major side effects included respiratory depression and intoxication. Minor catheter complications included primary placement failure, dislocation, disconnection, occlusion, loosened filter, and leakage. Major complications included focal neurologic deficit, epidural abscess, and hematoma.

Secondary outcome measures included the rate of other complications, length of stay in the intensive care unit (ICU) and hospital, duration of mechanical ventilation, and mortality (in-hospital and 30 days after discharge). Respiratory complications included pneumonia, need for intubation, atelectasis, acute respiratory distress syndrome, and the need of tracheotomy. Pneumonia was defined by presence of clinical symptoms (coughing, fever, and desaturation) requiring antibiotic treatment, regardless of a negative or positive culture. Diagnosis was confirmed by examination of a chest radiograph. Atelectasis was defined as collapse or incomplete expansion of pulmonary parenchyma confirmed on a chest radiograph or computed tomography scan. Acute respiratory distress syndrome was defined by severe hypoxemia with a PaO_2_/FIO ratio smaller than 100 mmHg.

### 2.4. Statistical Analysis

Data were described using frequencies and percentages for dichotomous and categorical variables, mean and standard deviation (SD) for normally distributed continuous data, and median and interquartile range (IQR) for non normally distributed continuous data.

To assess possible rib fracture characteristics independently associated with epidural analgesia failure, a multivariable logistic regression was performed. Subgroup analysis was performed for success of epidural analgesia on in-hospital outcome measures. Statistical significance was defined as *p* < 0.05. All statistical analyses were performed using Stata® 13.0 (StataCorp LP, College Station, TX, USA).

## 3. Results

### 3.1. Patients

**Figure 1:**
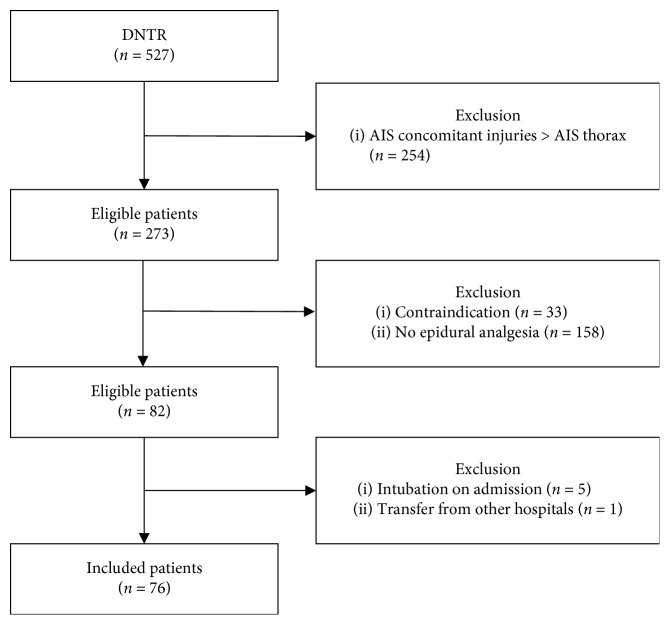
Flow chart representing the selection of the included patients.

A total of 527 patients were identified with the Dutch National Trauma Registry. Ultimately, 76 patients who received epidural analgesia were included in this study (Figure 1).

The included patients had a mean age of 58 (SD 14) years and were predominantly male (*n*=61, 80%). The median ISS was 14 (IQR 10-17) (Table 1). The mean number of rib fractures was 7 (SD 3), bilateral fractures occurred in 15 patients (20%), and 12 patients (24%) had a flail segment. Sixty-five patients (86%) had one or more fractured rib(s) in the upper thorax (costae 1 to 4), 74 patients (97%) in the middle thorax (costae 5 to 8), and 38 patients (50%) in the lower thorax (costae 9 to 12). Operative rib fixation was performed in 28 patients (37%) (Table 2).

Sixty-five patients (86%) received an epidural catheter primarily upon time of admission. In 11 patients (14%), catheter placement occurred secondarily during admission since sufficient pain control could not be achieved.

### 3.2. Efficacy of Epidural Analgesia

**Figure 2:**
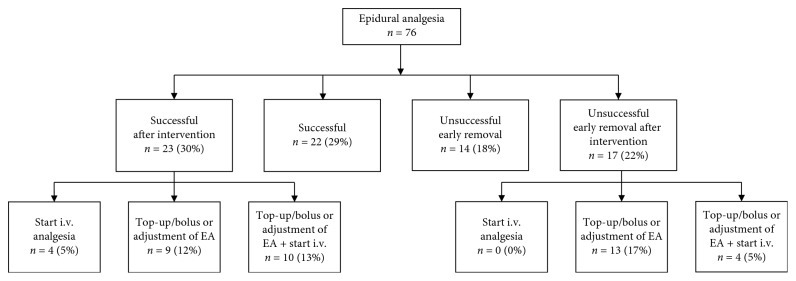
Flow chart representing the efficacy of epidural analgesia in patients with traumatic rib fractures.

As demonstrated in the flowchart in Figure 2, epidural analgesia was successful in the first 5 days in a total of 45 patients (59%). In 22 patients (29%), no intervention was needed, and in 23 patients (30%), an additional intervention was needed, which included administration of intravenous morphine in 4 patients (5%), an epidural top-up in 9 patients (12%), or a combination in 10 patients (13%). In 14 patients (18%), epidural analgesia was terminated before day 5 due to insufficient sensory blockade (*n*=4), medication-related side effects (*n*=4), and catheter-related complications (*n*=6). In 17 patients (22%), the epidural catheter was removed after one or multiple additional interventions due to insufficient pain control.

### 3.3. Side Effects and Complications

Medication-related side effects and catheter-related complication were encountered in 37 patients (49%) (Table 3). Minor medication-related side effects were reported in 28 patients (37%) and included nausea (*n*=10, 13%), pruritus (*n*=10, 13%), and hypotension with need of vasopressin support (*n*=7, 9%). One patient (1%) experienced a major side effect due to morphine intoxication with severe systemic effects, most likely because of coadministration of transdermal fentanyl. Minor catheter-related complications occurred in 9 patients (12%) and included primary placement failure (*n*=2, 3%), accidental dislocation (*n*=1, 1%), disconnection (*n*=3, 4%), occlusion (*n*=1, 1%), loosened filter (*n*=1, 1%), and leakage (*n*=1, 1%), and in one patient (1%), epidural medication was administered intravenously. No major complications occurred.

The epidural catheter was removed in only 5% of all patients due to one of the medication-related side effects, and in only 8% of all patients due to a catheter-related complication. All other medication-related side effects could be remedied by adjusting the medication.

### 3.4. Additional Analyses

A multivariable analysis was performed to identify rib fracture characteristics that were independently associated with epidural analgesia failure. The following rib fracture-related characteristics were included in our analysis: number of rib fractures, bilateral involvement, dislocation, first rib involvement, presence of dorsal fracture(s), and location of fractures (upper, middle, or lower part of thorax). No rib fracture characteristics appeared to be independently associated with epidural analgesia failure.

Table 4 shows the in-hospital outcomes stratified by the success rate of epidural analgesia. Two patients died in the group of unsuccessful epidural analgesia. One patient died in the ICU due to sepsis with multiorgan failure, and in one patient, the probable cause of death was a bilateral pneumonia. There were no further differences between the in-hospital outcome measures and success rate of epidural analgesia.

## 4. Discussion

International guidelines recommend epidural analgesia in patients with traumatic rib fractures. However, the evidence regarding the effects and safety of epidural analgesia remains inconclusive [9]. The aim of this study was to report on the success rate of epidural analgesia in patients with multiple rib fractures in the current practice. Epidural analgesia was successful in 59% of patients. Nonetheless, more than half of these patients needed additional interventions to achieve sufficient pain control. Epidural-related minor complications or side effects occurred in 49% of patients; however, this ultimately led to catheter removal in only 10% of all cases.

Previous studies on epidural analgesia after different types of surgery, reported incidence rates of epidural analgesia failure ranging from 13% to 47% [20]. A study by Ready included 25,000 patients who received postoperative epidural analgesia, reporting a failure rate of 32% in thoracic epidural analgesia and 27% in lumbar epidural analgesia. Similar to our findings, the most common reasons for epidural failure reported in the literature are unsatisfactory analgesia- or catheter-related complications such as early catheter dislodgment, leakage, or occlusion [24, 25].

About half of the patients in our study had complications or side effects after epidural analgesia. The majority were minor medication-related side effects such as pruritus, nausea, and hypotension. Other complications reported in the literature include bradycardia, respiratory depression, or decreased consciousness, and catheter-related complications such as epidural hematoma or abscess [26]. In our study, an opioid intoxication due to administration of both epidural and transdermal opioids was encountered in one patient. The incidence of catheter-related complications was 12% in this study, which is similar to the reported incidences in the current literature [27]. Ultimately, this resulted in removal of the epidural catheter in 8% of all cases. Therefore, it must be taken into account that risk of failure of the epidural catheter placement is an important contributing factor on the overall success rate.

The question whether epidural analgesia is beneficial over other analgesic modalities in patients with traumatic rib fractures is debatable [9, 17, 28]. A large multicenter retrospective cohort study of Gage et al. demonstrated a significant reduction in mortality in patients with multiple rib fractures who received epidural analgesia [10]. Similar findings were reported by Flagel et al., who examined the use of epidural analgesia in patients with multiple rib fractures, using the National Trauma Databank [1]. In a randomized controlled trial, Bulger et al. compared the effect of epidural analgesia with intravenous analgesia in patients with more than three rib fractures [6]. They concluded that epidural analgesia resulted in a decrease of incidence of pneumonia and duration of mechanical ventilation. However, they remarked that the feasibility of this analgesic modality is limited by numerous contraindications. In contrast, a recent matched-cohort study of McKendy et al. showed that patients with one or more fractured ribs who received epidural analgesia were associated with higher rates of respiratory complications and an increased hospital length of stay compared to patients who received other analgesic interventions [19]. They stated that possible explanations for a failed application of epidural analgesia were lack of experience with the use of epidural analgesia and inability of early mobilization. In response to this matched-cohort study of McKendy et al. [19], Amaral Saxe and Jensen performed the same analyses on a similar-sized cohort using a database at their institution. However, they found the opposite outcomes and reported a significant reduction in mortality in favor of patients receiving epidural analgesia [29].

In a recent systematic review and meta-analysis of both observational studies and randomized controlled trials, effects of epidural analgesia were compared with other analgesic modalities in patients with one or more traumatic rib fractures. Nineteen studies were included, representing a total of 2801 patients. This study showed that epidural analgesia provided better pain relief than other analgesic modalities, although few studies reported on pain scores. No beneficial effects from epidural analgesia could be demonstrated for the outcome measures hospital and intensive care unit length of stay, duration of mechanical ventilation, respiratory complications, and mortality [28].

Several difficulties are associated with the use of epidural analgesia in patients with traumatic rib fractures that are insufficiently highlighted in current practice while important information for decision-making. According to a recent systematic review, incidences of epidural-related complications are poorly reported [28]. Also, there are insufficient data on failure rates, need for additional interventions (e.g., epidural top-ups), duration of sufficient epidural analgesia, and need for additional (escape) medication. Furthermore, patients with multiple rib fractures are often polytrauma patients with concomitant injuries making these patients frequently not eligible for epidural analgesia. Moreover, it remains unclear to what extent outcomes are affected by other concomitant injuries.

This study had several limitations. First, due to the retrospective nature of this study, results are subject to missing data and underreporting. Pain perspective is an important outcome measure; however, this could not be accurately assessed due to missing data. Therefore, we could not provide an overall presentation of the pain scores of this cohort. Additionally, there were insufficient data to calculate the daily used intravenous morphine. However, the number of patients who received additional intravenous opioids has been described. Second, patients were selected using the AIS thorax which might have resulted in a specific subgroup of patients limiting generalizability of the study results. Third, the number of included patients was relatively small. So, although we did not identify a significant difference in mortality between patients with or without successful epidural analgesia, it must be considered that this might be due to a limited power.

Finally, the available literature reporting on the efficacy of epidural analgesia in patients with multiple traumatic rib fractures remains scarce; therefore, this study contributes to the current literature and discussion of optimal management of these patients.

## 5. Conclusion

Epidural analgesia was successful in 59% of patients; however, 30% needed additional analgesic interventions. As about half of the patients had epidural-related complications, it remains debatable whether epidural analgesia is a sufficient treatment modality in patients with multiple rib fractures. Future research could focus on other regional analgesic modalities that are more effective and less susceptible to complications.

## Figures and Tables

**Table 1:** Baseline characteristics of patients with epidural analgesia for multiple traumatic rib fractures.

Variable	Total *n*=76
Age (mean ± SD)	58 ± 14
Sex (*n*, %)	
Male	61 (80)
Female	15 (20)
Trauma mechanism (*n*, %)	
Motor-vehicle	21 (28)
Bicycle	17 (22)
Fall	19 (25)
Assault	1 (1)
Others	18 (24)
ISS (median, IQR)	14 (10–17)
AIS (median, IQR)	
Head	0 (0-0)
Face	0 (0-0)
Chest	3 (3-3)
Abdomen	0 (0-0)
Extremity	0 (0–2)
External	1 (0-1)
GCS (median, IQR)	15 (15-15)
Concomitant injuries (*n*, %)	
Lung contusion	25 (33)
Pneumothorax	30 (39)
Hematothorax	8 (11)

AIS: Abbreviated Injury Scale; GCS: Glasgow Coma Scale; ISS: Injury Severity Score; IQR: interquartile range; *n*: number; SD: standard deviation.

**Table 2:** Fracture characteristics.

Variable	Total *n*=76
Number of rib fractures (mean ± SD)	7 ± 3
Bilateral rib fractures (*n*, %)	15 (20)
Location rib fracture (*n*, %)	
Costae 1–4	65 (86)
Costae 5–8	74 (97)
Costae 9–12	38 (50)
First rib fracture (*n*, %)	21 (28)
Flail segment (*n*, %)	12 (24)
Displacement (*n*, %)	31 (41)
Dorsal fracture (*n*, %)	54 (71)
Rib fixation (*n*, %)	28 (37)

IQR: interquartile range; *n*: number; SD: standard deviation.

**Table 3:** Side effects and complications.

Variable	Total *n*=37
Medication related (*n*, %)	
Hypotension	7 (9)
Nausea	10 (13)
Pruritus	10 (13)
Intoxication	1 (1)

Catheter related (*n*, %)	
Primary placement failure	2 (3)
Dislocation	1 (1)
Disconnection	3 (4)
Occlusion	1 (1)
Loosened filter	1 (1)
Leakage	1 (1)
Focal neurologic deficits	0 (0)

*n*: number.

**Table 4:** In-hospital outcome measures in patients with successful or unsuccessful epidural analgesia.

Variable	Successful *n*=45	Unsuccessful *n*=31
Hospital length of stay (median, IQR)	10 (7–12)	10 (8–17)
Intensive care length of stay (median, IQR)	0 (0–0)	0 (0–0)
Duration of mechanical ventilation (median, IQR)	0 (0–0)	0 (0–0)
Respiratory complications (*n*, %)		
Pneumonia	6 (13)	3 (10)
Atelectasis	4 (9)	2 (6)
ARDS	0 (0)	1 (3)
Mortality (*n*, %)		
During admission	0 (0)	2 (6)
Postdischarge 30 days	0 (0)	0 (0)

ARDS: acute respiratory distress syndrome; IQR: interquartile range; *n*: number; SD: standard deviation.

## Data Availability

The data used to support the findings of this study are restricted by the Medical Research Ethics Committee of the UMC Utrecht in order to protect patient privacy. Data are available from the University Medical Center Utrecht for researchers who meet the criteria for access to confidential data. Requests for access to these data should be made to the Medical Research Ethics Committee of the UMC Utrecht.
